# Grand Challenges: Improving HIV Treatment Outcomes by Integrating Interventions for Co-Morbid Mental Illness

**DOI:** 10.1371/journal.pmed.1001447

**Published:** 2013-05-21

**Authors:** Sylvia Kaaya, Eddy Eustache, Ilana Lapidos-Salaiz, Seggane Musisi, Christina Psaros, Lawrence Wissow

**Affiliations:** 1 School of Medicine, Muhimbili University of Health and Allied Sciences, Dar es Salaam, Tanzania; 2 Zanmi Lasante/Partners In Health, Central Plateau, Haiti; 3 United States Agency for International Development, Office of HIV/AIDS, Washington D.C., United States of America; 4 School of Medicine, Makerere University, Kampala, Uganda; 5 Department of Psychiatry, Massachusetts General Hospital, Boston, Massachusetts, United States of America; 6 Center for Mental Health in Pediatric Primary Care, John Hopkins University, Baltimore, Maryland, United States of America

## Abstract

In the fourth article of a five-part series providing a global perspective on integrating mental health, Sylvia Kaaya and colleagues discuss the importance of integrating mental health interventions into HIV prevention and treatment platforms.

*Please see later in the article for the Editors' Summary*

**Summary Points**

- Clinical depression, alcohol abuse, and HIV-associated neurocognitive disorders are highly prevalent in people living with HIV and have negative consequences for HIV treatment outcomes.
- Effective interventions exist for recognition and treatment of these co-morbid mental disorders and can be implemented successfully by trained non- specialized providers.
- “Task-sharing” requires supportive supervision, monitoring, and feedback to inform quality improvement in comprehensive HIV/AIDS services providing mental health care.
- Multidisciplinary collaboration, coordination, and communication on common concerns are imperative for HIV services that integrate mental health care.
- Integration of mental health interventions into HIV prevention and treatment platforms can reduce opportunity costs of care and improve treatment outcomes.
- This paper is the fourth in a series of five articles providing a global perspective on integrating mental health.

*This is one article in a five-part series providing a global perspective on integrating mental health.*

## Introduction

Mental, neurological, and substance use (MNS) disorders occur frequently in patients with HIV and are associated with negative outcomes, including reduced adherence to antiretroviral medications (cART), and diminished quality of life. A review of PubMed and PsychInfo from 2001 to 2012 revealed a dearth of evaluated mental health services in HIV primary care, particularly in low- and middle income countries. Available findings suggest, however, that opportunities do exist in HIV primary care to integrate interventions for recognition and treatment of depression and alcohol use disorders and prevention of HIV neurocognitive disorders (HAND).

Among patients receiving treatment for HIV, MNS disorders may occur at rates that exceed those of physical co-morbidities [1]. Depression is twice as common in people living with HIV (especially when symptomatic) than in uninfected individuals [2],[3]. In addition, alcohol use is frequent in persons receiving treatment for HIV; in one sub-Saharan African study, current and hazardous alcohol use were reported as 27% and 3.0%, respectively, with adverse effects on cART adherence [4],[5]. HAND prevalence ranges from 20% to 56% worldwide, and is especially high in older patients and those with advanced immuno-suppression. In low-income countries, HAND is compounded by poverty, opportunistic central nervous infections, and assessment challenges [6],[7].

A variety of mechanisms links MNS disorders to HIV disease. First, the social conditions under which most patients with HIV live (e.g., limited employment, housing and food insecurity, exposure to stigma, and fear of serostatus disclosure) contribute to the development and exacerbation of MNS disorders [8]. MNS disorders, in turn, are associated with greater suffering, including poorer psychological adjustment to a chronic, progressive and life-threatening illness; lower quality of life [9],[10]; worse HIV treatment adherence and outcomes [5],[6]; and an increased risk of HIV transmission [11]. Second, HIV co-morbid substance use disorders can influence HIV transmission by increasing vulnerability to sexual exploitation and impairing the judgment required to engage in safe sexual practices [11],[12]. Third, HIV directly affects the central nervous system (CNS), with increasing evidence of long-term cognitive effects that may, despite achievement of non-detectable viral load, not be reversible with currently available cART [13]. HIV also puts individuals at risk for acquiring other infectious and non-infectious conditions that affect the CNS–including malaria, tuberculosis, and lymphomas —and further impair CNS function [7].

The need to redesign health systems to integrate care for MNS disorders with other chronic disease care was identified as one of the Grand Challenges in Global Mental Health (GCGMH) [14]. Although robust evidence for occurrence of HIV and MNS disorder co-morbidities exists, less evidence regarding implementation of programs that integrate their care within HIV treatment is available, particularly in settings with generalized epidemics. At a minimum, packages of care for selected MNS disorders should be included when scaling-up HIV primary care prevention and treatment services. This paper is the fourth in a five-part series providing a global perspective on integrating mental health.

## The Grand Challenge of Integrating Mental Health into HIV Primary Care

Standardized cART regimens, toxicity management, monitoring, and simplified clinical decision making have facilitated access to HIV prevention, treatment, and care in general medical care and HIV primary care, particularly in low- and middle-income countries [15]. The potential to also treat co-morbid disorders is supported by robust evidence of positive treatment outcomes when depression and alcohol abuse screening and treatment are integrated into primary care. Meta-analyses of studies of collaborative management of depression in general primary care show improvement in baseline depressive symptoms (standardized mean difference = 0.34), adherence to depression treatment (odds ratio [OR] = 2.22) and response to treatment (OR = 1.78) [16], with strong support for these effects across a broad range of primary care settings, including in low-resourced settings [17],[18]. Additional evidence demonstrates the cost-effectiveness of integrating interventions for depression and alcohol abuse in primary care [19],[20]. Improving the understanding of core processes and components of such integration is important, however, to ensuring its success. The focus of this discussion are the challenges to integrating mental health services into primary HIV care, with an emphasis on studies in low- and middle-income countries, using the summary principles of the GCGMH: use of a life course approach, use of evidence-based interventions, understanding environmental influences, and use of system-wide approaches to alleviate suffering.

### Use a Life Course Approach

In children, as with adults, early initiation of HIV treatment, before prolonged periods of elevated viral load, may reduce the incidence of static cognitive problems that negatively affect school and vocational achievement [21],[22]. Awareness among HIV providers of long-term cognitive risks may promote higher surveillance and earlier decisions to start cART. A lack of information on the potential adverse effects of early cART treatment remains, however. While cART has been shown to arrest neurocognitive impairment, even some well-treated children do not seem to attain the developmental status of their peers [23]. Although the risks of intrauterine exposure to medications used to prevent vertical transmission remain unknown, early prevention of HIV offers the best opportunity to avoid life-long HAND morbidity. Prevention opportunities (e.g., PMTCT) that are available within the context of HIV treatment and general medical care include HIV testing and initiation of cART during pregnancy, elective caesarean or minimally invasive assisted deliveries and exclusive or deferred breast-feeding when it is affordable and safe. These interventions have reduced the rates of vertical HIV transmission in both high- and low-resourced settings [24],[25].

Efforts to engage women in PMTCT may be undermined, however, by co-occurring mental health problems. Excessive alcohol consumption may interfere with the use of preventive health care and decisions to test for HIV during pregnancy. Post-delivery depression can reduce mothers' participation in early infant HIV diagnosis and infants' adherence to short course antiretroviral prophylactic medication [26]. This interference further makes the case for integration of mental health services within HIV prevention and care.

Unfortunately, PMTCT services are often established as vertical programs, lacking sufficient integration even with maternal, neonatal, and child health programs, which share similar goals and provide a basic platform and infrastructure for effective and sustainable delivery of HIV services. In some low-resourced settings, however, it has been feasible to include expanded PMTCT as an integral component of antenatal care by establishing on-site cART services as well as training maternal and child health nurses to implement simplified integrated protocols [27]. Ancillary services, such as caregiver social support, have the potential to influence adherence to cART and treatment plans for mothers and children. They could also support children to cope with and adjust to HIV, with potential positive effects on behavioral and emotional symptoms, some of which are associated with neurocognitive harms [22],[28].

### Use Evidence-based Interventions when Integrating HIV and MNS Disorders

Evidence-based mental health interventions are available that can be delivered in non-specialist mental health settings, making them potentially ideal for HIV care settings or for primary care. Effective screening instruments to facilitate diagnosis and pharmacological and psychological treatments exist for depression [29],[30]. Empirically supported psychosocial treatments for depression, including cognitive behavioral therapy (CBT), interpersonal psychotherapy (IPT), and problem-solving therapy (PST), have been studied in the context of HIV in resource-rich [30] and -limited countries [29],[31],[32]. Such therapies can be delivered to individuals or groups, with the number of sessions planned based on the level of psychopathology and treatment goals. Many protocols use between 2 and 20 sessions. Intervention outcomes vary and include reduced severity of depressive symptoms, increased positive coping, facilitation of support networks, and increased self-esteem [30],[32]. Good outcomes have been demonstrated when non-specialized primary health care workers [33] and trained lay providers [32] deliver interventions. CBT and PST can also be used to address problems or goals associated with health-related behavior change, such as adherence to antiretroviral therapy [34].

Screening for hazardous alcohol use combined with brief interventions (SBI) includes providing feedback on assessed levels of drinking; communicating potential deleterious effects; recommending reducing levels of alcohol consumption; and, where necessary, referring for specialized care. Reviews show that when SBI is delivered by trained primary care workers, the proportion of patients drinking at moderate or safe levels increases by 10% to 19% compared with controls [35]. Systematic reviews show that one or two follow-up SBI sessions are more effective than single sessions, and report sustained reductions in the levels of alcohol consumption assessed at 6 and 12 months [35]. Problem drinkers show a 23% to 26% reduction in mortality [36]. These improvements are modest but notable relative to their cost and potential widespread availability. Integrating SBI into HIV care also provides an opportunity for assessing incremental benefits in HIV treatment outcomes due to improvements in cART adherence, fewer opportunistic infections, and fewer HIV risk behaviors as problem drinking decreases.

The diagnosis of HAND in primary care is complicated by a lack of instruments validated across cultures and literacy levels [7]. The International HIV Dementia Scale, which translates well across cultures, assesses more motor-based skills and is not as useful for less severe levels of impairment [7]. Milder levels of neurocognitive impairment in children and adults may best be detected through interviews with partners, close family members, and patients to examine changes in social, home, school, or work functioning over time or, for children, to make comparisons with same-age peers. Early recognition of HAND, particularly in children, provides an opportunity for rehabilitative interventions. Computerized cognitive rehabilitation therapy, based on an intervention developed to improve working memory and executive function, is one still experimental intervention that is feasible in a low-resourced setting and demonstrates neuropsychological and psychosocial benefits in children with HAND [37]. While antiretroviral drugs vary in their ability to penetrate the brain, it is not yet clear if treatment with cART with higher compared to lower brain penetrance can prevent the more subtle but clinically significant forms of HAND. Some evidence exists that higher penetration cART improves survival in children, whether or not they develop HAND [38]. Moreover, there is potential to task-shift by involving existing lay counselors working in basic HIV treatment settings in low-resourced contexts in the provision of ancillary services, such as helping parents recognize that affected children may need additional help with school work or more step-wise explanation of household tasks/chores to prevent additional educational, social, and emotional problems.

### Understand Environmental Influences

The treatment and preventive interventions described thus far should be implemented in a given health system and social context. Successful adaptation and integration of effective interventions require understanding of a system's resources as well as the acceptability of the intervention to the target population and providers. Just as important are the everyday life stressors influencing families' abilities to manage illness that form part of the overall context of care needing programmatic attention.

Patients who attend collocated multidisciplinary services experience improved access to cART and retention in care in high-resourced contexts [38]. While evidence from low-resourced settings is limited, collocating targeted mental health services may be feasible and beneficial. For example, in Haiti, a program for HIV-affected youth and their caregivers' targeted issues specific to living with HIV in the community and strengthening support within families. Components of a social cognitive theory-based intervention, Project TALC (Teens and Adults Learning to Communicate), were adapted to both meet specific local requirements for acceptability and accessibility and incorporate culturally congruent conceptions of wellness and healing. The adaptation process, led by an indigenous psychologist, addressed both health care provider priorities and caregivers' concerns about children's school performance and behavioral problems. The briefer adapted program focused on survival of caregivers, reducing distress, and improving caregiver-child communication. Initial caregiver-only support group sessions were followed by separate and joint parent-child groups that addressed self-care, HIV prevention, stigma awareness, strategies for supporting children, and building active listening and problem solving skills. Post intervention, HIV-affected youth and their caregivers experienced decreased psychological symptoms, improved psychosocial functioning, and increased perceived ability to cope and hope for the future; caregivers reported less HIV-related stigma [31].

The Haiti example reiterates the importance of collaborating with target populations during adaptation and testing of evidence-based mental health interventions to ensure acceptance and success of integrated HIV and mental health care. Subsequent steps may include joint planning by mental health and HIV primary care providers to refine interventions informed by test findings, develop operational and monitoring tools, and train and re-orient providers for seamless integration of psychosocial care components at practice and health system levels.

### Use System-wide Approaches to Alleviate Suffering

Many HIV care and treatment programs that have received considerable funding as vertical programs increasingly face the demand to integrate additional services or be integrated into the general medical sector. In this context, integrating care for MNS disorders can be justified in a number of ways. First, individuals tend to seek physical rather than psychological causes for difficulties associated with chronic disease [39]. In some cases, onset of a mental disorder, e.g., abrupt changes in mood may be the first sign of a new or worsening medical problem. The ability to conduct a thoughtful, comprehensive evaluation that includes mental health may lead to more rapid and appropriate treatment for both the mental health and somatic problems. Second, individuals with chronic conditions encounter high opportunity costs to maintain their health, especially in low-resourced countries where the costs of transportation to care sites and missed work – even when care itself is free – can exceed a family's available resources. Integrated services help keep these costs to a minimum by potentially reducing the number of visits required or care sites that one might need to attend. Third, integrated services may increase access to services by minimizing boundaries across different systems of care, ease navigation of the health systems in patients with mental or cognitive disabilities, and reduce barriers to treatment associated with stigma. Mental health care delivered at general medical settings is more discrete and may in itself help reduce stigma by normalizing MNS disorders as common complications of the chronic condition.

A small but growing number of studies suggest that care for mental disorders can be integrated with general medical care in low-resourced countries. Some models are based on collocating mental health specialists while others focus on “task-sharing ”or “task-shifting,” i.e., training lay workers or improving the mental health skills of generalists [40]. Even collocation models, however, rely on the development of interventions for mental disorders that can be delivered over a relatively brief period and without great cost. While interventions adapted for the HIV setting show promise [28],[31],[32] well-designed and powered prospective studies are needed in the future.

## Steps to Implementing Integrated HIV and Mental Health Primary Care

Four key areas are important when considering integrating services for MNS disorders into HIV primary care. First, integration requires creating a norm among both patients and providers that treating MNS disorders is a legitimate part of HIV care. The use of brief screening instruments for such disorders can create this signal and offer providers guidance in identifying patients with possible problems. Established methods for tailoring instruments locally for detection and tracking of MNS disorders may help to increase cross-cultural validity. Instruments that have been useful in HIV-care settings for depression and alcohol abuse are summarized in Table 1. They can be completed independently, or with a health care worker's assistance, and can be interpreted with minimal training.

**Table 1:** Standardized Depression and Alcohol Use Disorder Screening Tools, Training Required, and Administration Time.

Instrument	Languages Available	Training Required	Other
Center for Epidemiologic Studies Depression Scale (CES-D)	English and more than 40 additional languages	None/minimal	Approximately 10 minutes to complete and 5 minutes to score. Contains fewer somatic items than other depression self-report measures. ≥16 is used as cut-off score for likely depression.
Patient Health Questionnaire 9 (PHQ-9)	Arabic, Assamese, Chinese (Cantonese, Mandarin), Czech, Dutch, Danish, English, Finnish, French, French Canadian, German, Greek, Gujarati, Hindi, Hebrew, Hungarian, Italian, Malay, Malayalam, Norwegian, Oriya, Polish, Portuguese, Russian, Spanish, Swedish, and Telugu	None/minimal	Approximately 5 minutes to complete and score. Designed for use in primary care settings. ≥10 is used as the cut-off for likely depression. Includes a suicide screening question.
Hopkins Symptom Checklist Depression Scale	English, Bosnian, Cambodian, Croatian, Japanese, Laotian, and Vietnamese	None/minimal	Approximately 10 minutes to administer and score; scoring requires computing an average. >1.75 is used as a cut-off for likely depression.
Alcohol Use Disorders Identification Test (AUDIT)	English, Catalan and Spanish, Dutch, French, Portuguese, Russian, Slovenian, Swedish	None/minimal	Approximately 4 minutes to administer and score; >7 warrants further assessment; scores provide 4 levels of AUD severity
CAGE Questionnaire	English and several other languages because four items are straightforward in translation	None/minimal	Approximately 1–2 minutes to administer and score; ≥2 is used to assess for alcohol dependence

Second, integration requires actions directed at people seeking services, local providers, and regional or national health care systems [41]. Persons seeking services should be viewed as partners whose needs for medical care, social inclusion, and psychological support will be addressed through the integrated services. At the local health system level, collaboration between HIV and mental health services should include co-training providers, selecting task-shared activities, sustaining ongoing supervision, and establishing guidelines for referral for specialist consultation. At the regional and national levels, resource sharing, planning for human resource needs, establishing collaborative relationships across sectors, implementing policies and strategic planning, and monitoring integration and implementation scale up are appropriate. Table 2 outlines characteristics of systems and services integration and anticipated outcomes. Integration requires careful consideration of disease processes and service delivery practices for both HIV and mental disorders. Core mental health intervention components that can be delivered through PMTCT and HIV care and treatment platforms should be identified. This identification requires collaboration between HIV service providers and existing mental health specialists so that procedures for joint care can be developed. Specifically, teams should determine what kinds of interventions (screening, treatment, and referral) will be offered at various HIV care facilities (e.g., cART clinics, voluntary counseling and testing centers, PMTCT services, community/home-based care and support services, and within spiritual and traditional healing systems).

**Table 2:** Integrating Mental Health Services into Primary HIV Care For Women: The Whole Life Project.

System Integration Characteristics	Service Integration Characteristics	Integration Outcomes
Inter-domain coordination For example: How can relevant managers coordinate across mental health and HIV services?	Shared commitment to comprehensive care, prevention, and early intervention	Collaborative development of a comprehensive service packages (client's physical, psychological, social and cultural dimensions acknowledged) in a context comprising family, neighborhoods, and larger institutions aimed at prevention and early interventions
Collaborative planning, programming, resources sharing, and communication	Collocation of services and staff	Accessible and seamless service delivery, perceived unified and non-redundant by users
Administrative sanctioning of integration	Administrative and programmatic activities that reflect integration, including joint planning, resource and information sharing; staff cross-training and team building	Effective and shared patient management, case referrals, and follow-up procedures; joint supportive supervision and outcome monitoring, quality assessment, and re-planning for service quality improvements

Source: Adapted from Dodds et al. [42].

Third, consensus on the cadre of providers should be reached. Where mental health specialists are scarce, building and sustaining appropriate capacity among other kinds of providers should include supportive supervision to reduce burn-out risks and provide ongoing training, outcome monitoring, and quality assessment and improvement support. Parallel and continual development of specialists with an awareness of the local environment may be needed. Mental health problems in the context of HIV can be caused by a variety of medical conditions (infectious, vascular, or toxic), the nature of which may vary from region to region. In children, these disorders can occur alongside developmental problems. Providers require diagnostic skills and specific training in the recognition of the MNS disorders most likely to occur in the populations they serve. Moreover, they should be sensitive to local expressions of social exclusion and discrimination that often accompany diagnoses of both HIV infection and MNS disorders. Table 3 outlines the necessary components of mental health interventions and key features for inclusion in HIV care platforms.

**Table 3:** Recommendations for Delivering Mental Health Services in HIV Care.

Strengthening Mental Health Packages of Care	Mental Health Components for HIV Care
• Establish sustainable training and supervision models to maintain proficiency in mental health intervention delivery among non-specialists• Utilize evidence-based interventions after adequate situational analysis/needs assessment• Utilize a collaborative approach between HIV and mental health providers to adapt interventions to the cultural context as needed• Ensure adequate monitoring, evaluation, and feedback of information to generate intervention improvements• Educate communities about mental disorders and mental health to increase community uptake of interventions• Ensure effective referral and linkage systems to mental health specialist consultation when needed• Ensure availability of antidepressant, antipsychotic, and anticonvulsant medications	• Strengthen HIV policies, guidelines, and curricula by including relevant mental health service components• Increase access to interventions for early recognition of depression, alcohol abuse, and HAND among persons accessing HIV care• Provide cost-effective brief psychological interventions for depression and alcohol abuse• Re-enforce strategies for HIV screening, earlier access to cART, and early recognition and treatment of opportunistic infections to prevent HAND in children and adults• Monitor the effect of the mental health interventions on adherence to HIV care• Strengthen existing psychosocial interventions for social support• Strengthen systems for supply of antidepressant and antipsychotic medications in HIV primary care• Provide interventions as seamlessly integrated, routine components of care at the point of service for persons accessing HIV care

HAND, HIV-associated neurocognitive disorder.

Integration of HIV primary care and mental health provides the opportunity for both improving early detection of MNS co-morbidities with HIV infection and implementing effective interventions appropriate for existing health, family, and community resources. The evidence for the need is clear, and the evidence base for interventions suggests that it is feasible in low-resourced settings. Ultimately, successful integration of HIV and mental health services will require shared commitment of providers and policymakers along with collaborative learning to address remaining challenges.

## Abbreviations

cART: antiretroviral medications
CBT: cognitive behavioral therapy
CNS: central nervous system
GCGMH: Grand Challenges in Global Mental Health
HAND: HIV neurocognitive disorders
IPT: interpersonal psychotherapy
MNS: mental, neurological, and substance use disorders
PMTCT: prevention of maternal to child transmission of HIV
PST: problem-solving therapy
SBI: screening for hazardous alcohol use combined with brief interventions

## References

[1] Goulet JL, Fultz SL, Rimland D, Butt A, Gibert C, et al (2007) Aging and infectious diseases: do patterns of comorbidity vary by HIV status, age, and HIV severity? Clin Infect Dis 45 12: 1593–1601. 18190322 10.1086/523577 PMC3687553

[2] Maj M, Janssen R, Starace F, Zaudig M, Satz P, et al (1994) WHO neuropsychiatric AIDS study, cross-sectional Phase-I - Study design and psychiatric findings. Arch Gen Psychiatry 51 1: 39–49. 8279928 10.1001/archpsyc.1994.03950010039006

[3] Owe-Larsson B, Sall L, Salamon E, Allgulander C (2009) HIV infection and psychiatric illness. Afr J Psychiatry 12: 115–128. 10.4314/ajpsy.v12i2.43729 19582313

[4] Jaquet A, Ekouevi DK, Bashi J, Aboubakrine M, Messou E, et al (2010) Alcohol use and non-adherence to antiretroviral therapy in HIV-infected patients in West Africa. Addiction 105 8: 1416–1421. 20528816 10.1111/j.1360-0443.2010.02978.x PMC4458514

[5] Mayston R, Kinyanda E, Chishinga N, Prince M, Patel V (2012) Mental disorder and the outcome of HIV/AIDS in low-income and middle-income countries: a systematic review. AIDS 26 Suppl 2: S117–S135. 23303434 10.1097/QAD.0b013e32835bde0f

[6] Hinkin CH, Castellon SA, Atkinson JH, Goodkin K (2001) Neuropsychiatric aspects of HIV infection among older adults. J Clin Epidemiol 54 Suppl 1: S44–S52. 11750209 10.1016/s0895-4356(01)00446-2 PMC2864032

[7] Robertson K, Liner J, Heaton R (2009) Neuropsychological Assessment of HIV-Infected Populations in International Settings. Netherlands: Springer. Neuropsychology Review pp. 232–248. 10.1007/s11065-009-9096-z PMC2690834 19455425

[8] Collins PY, Holman AR, Freeman MC, Patel V (2006) What is the relevance of mental health to HIV/AIDS care and treatment programs in developing countries? A systematic review. AIDS 20 12: 1571–1582. 16868437 10.1097/01.aids.0000238402.70379.d4 PMC2801555

[9] Patel R, Kassaye S, Gore-Felton C, Wyshak, Kadzirange, et al (2009) Quality of life, psychosocial health, and antiretroviral therapy among HIV-positive women in Zimbabwe. AIDS Care 21 12: 1517–1527. 20024731 10.1080/09540120902923055 PMC4431539

[10] Sherbourne CD, Hays RD, Fleishman JA, Vitiello B, Magruder KM, et al (2000) Impact of psychiatric conditions on health-related quality of life in persons with HIV infection. Am J Psychiatry 157 2: 248–254. 10671395 10.1176/appi.ajp.157.2.248

[11] Shuper PA, Neuman M, Kanteres F, Baliunas D, Joharchi N, et al (2010) Causal considerations on alcohol and HIV/AIDS–a systematic review. Alcohol Alcohol 45 2: 159–166. 20061510 10.1093/alcalc/agp091

[12] Crepaz N, Marks G (2002) Towards an understanding of sexual risk behavior in people living with HIV: a review of social, psychological, and medical findings. AIDS 16 2: 135–149. 11807297 10.1097/00002030-200201250-00002

[13] Heaton RK, Franklin DR, Ellis RJ, McCutchan JA, Letendre SL, et al (2011) HIV-associated neurocognitive disorders before and during the era of combination antiretroviral therapy: differences in rates, nature, and predictors. J Neurovirol 17 1: 3–16. 21174240 10.1007/s13365-010-0006-1 PMC3032197

[14] Collins PY, Patel V, Joestl SS, March D, Insel TR, et al (2011) Grand challenges in global mental health. Nature 475 7354: 27–30. 21734685 10.1038/475027a PMC3173804

[15] Gilks CF, Crowley S, Ekpini R, Gove S, Perriens J, et al (2006) The WHO public-health approach to antiretroviral treatment against HIV in resource-limited settings. Lancet 368 9534: 505–510. 16890837 10.1016/S0140-6736(06)69158-7

[16] Thota AB, Sipe TA, Byard GJ, Zometa CS, Hahn RA, et al (2012) Collaborative care to improve the management of depressive disorders: a community guide systematic review and meta-analysis. Am J Prev Med 42 5: 525–538. 22516495 10.1016/j.amepre.2012.01.019

[17] Patel V, Araya R, Chatterjee S, Chisholm D, Cohen A, et al (2007) Treatment and prevention of mental disorders in low-income and middle-income countries. Lancet 370 9591: 991–1005. 17804058 10.1016/S0140-6736(07)61240-9

[18] Simon G (2009) Collaborative care for mood disorders. Curr. Opin. Psychiatry 22 1: 37–41. 10.1097/YCO.0b013e328313e3f0 19122533

[19] Gilbody S, Bower P, Whitty P (2006) Costs and consequences of enhanced primary care for depression: systematic review of randomised economic evaluations. Br J Psychiatry 189: 297–308. 17012652 10.1192/bjp.bp.105.016006

[20] Kraemer KL (2007) The cost-effectiveness and cost-benefit of screening and brief intervention for unhealthy alcohol use in medical settings. Subst Abuse 28 3: 67–77. 10.1300/J465v28n03_07 18077304

[21] Van Rie A, Dow A, Mupuala A, Stewart P (2009) Neurodevelopmental trajectory of HIV-infected children accessing care in Kinshasa, Democratic Republic of Congo. J Acquir Immune Defic Syndr 52 5: 636–642. 19730268 10.1097/QAI.0b013e3181b32646 PMC2787989

[22] Steele RG, Nelson TD, Cole BP (2007) Psychosocial functioning of children with AIDS and HIV infection: review of the literature from a socioecological framework. J Dev Behav Pediatr 28 1: 58–69. 17353739 10.1097/DBP.0b013e31803084c6

[23] Heidari S, Mofenson LM, Hobbs CV, Cotton MF, Marlink R, et al (2012) Unresolved antiretroviral treatment management issues in HIV-infected children. J Acquir Immune Defic Syndr 59 2: 161–169. 22138766 10.1097/QAI.0b013e3182427029 PMC4825866

[24] Creese A, Floyd K, Alban A, Guinness L (2002) Cost-effectiveness of HIV/AIDS interventions in Africa: a systematic review of the evidence. Lancet 359 9318: 1635–1643. 12020523 10.1016/S0140-6736(02)08595-1

[25] Townsend CL, Cortina-Borja M, Peckham CS, de Ruiter A, Lyall H, et al (2008) Low rates of mother-to-child transmission of HIV following effective pregnancy interventions in the United Kingdom and Ireland, 2000–2006. AIDS 22 8: 973–981. 18453857 10.1097/QAD.0b013e3282f9b67a

[26] Peltzer K, Mlambo G (2010) Factors determining HIV viral testing of infants in the context of mother-to-child transmission. Acta Paediatr 99 4: 590–596. 20064134 10.1111/j.1651-2227.2009.01670.x

[27] Pfeiffer J, Montoya P, Baptista AJ, Karagianis M, Pugas Mde M, et al (2010) Integration of HIV/AIDS services into African primary health care: lessons learned for health system strengthening in Mozambique - a case study. J Int AIDS Soc 13: 3. 20180975 10.1186/1758-2652-13-3 PMC2828398

[28] Futterman D, Shea J, Besser M, Stafford S, Desmond K, et al (2010) Mamekhaya: a pilot study combining a cognitive-behavioral intervention and mentor mothers with PMTCT services in South Africa. AIDS Care 22 9: 1093–1100. 20824562 10.1080/09540121003600352 PMC4019405

[29] Patel V, Simon G, Chowdhary N, Kaaya S, Araya R (2009) Packages of care for depression in low- and middle-income countries. PLoS Med 6: e1000159 doi:10.1371/journal.pmed.1000159 19806179 10.1371/journal.pmed.1000159 PMC2747016

[30] Himelhoch S, Medoff DR, Oyeniyi G (2007) Efficacy of group psychotherapy to reduce depressive symptoms among HIV-infected individuals: a systematic review and meta-analysis. AIDS Patient Care STDS 21 10: 732–739. 17949272 10.1089/apc.2007.0012

[31] Smith Fawzi MC, Eustache E, Oswald C, et al (2012) Psychosocial support intervention for HIV-affected families in Haiti: implications for programs and policies for orphans and vulnerable children. Soc Sci Med 74 10: 1494–1503. 22444459 10.1016/j.socscimed.2012.01.022

[32] Chibanda D, Mesu P, Kajawu L, Cowan F, Araya R, Abas M (2011) Problem-solving therapy for depression and common mental disorders in Zimbabwe: piloting a task-shifting primary mental health care intervention in a population with a high prevalence of people living with HIV. BMC Public Health 11 1: 828. 22029430 10.1186/1471-2458-11-828 PMC3210104

[33] Himelhoch S, Ehrenreich M (2007) Psychotherapy by primary-care providers: results of a national sample. Psychosomatics 48 4: 325–330. 17600169 10.1176/appi.psy.48.4.325

[34] Crepaz N, Lyles CM, Wolitski RJ, Passin WF, Rama SM, et al (2006) Do prevention interventions reduce HIV risk behaviours among people living with HIV? A meta-analytic review of controlled trials. AIDS 20 2: 143–157. 16511407 10.1097/01.aids.0000196166.48518.a0

[35] Whitlock EP, Polen MR, Green CA, Orleans T, Klein J (2004) Behavioral counseling interventions in primary care to reduce risky/harmful alcohol use by adults: a summary of the evidence for the U.S. Preventive Services Task Force. Ann Intern Med 140 7: 557–568. 15068985 10.7326/0003-4819-140-7-200404060-00017

[36] Cuijpers P, Riper H, Lemmers L (2004) The effects on mortality of brief interventions for problem drinking: a meta-analysis. Addiction 99 7: 839–845. 15200579 10.1111/j.1360-0443.2004.00778.x

[37] Bangirana P, Allebeck P, Boivin MJ, John CC, Page C, et al (2011) Cognition, behavior and academic skills after cognitive rehabilitation in Ugandan children surviving severe malaria: a randomised trial. BMC Neurol 11: 96. 21816079 10.1186/1471-2377-11-96 PMC3171314

[38] Wright E (2011) Neurocognitive impairment and neurocART. Curr Opin HIV AIDS 6 4: 303–308. 21546833 10.1097/COH.0b013e3283477c46

[39] de Ridder D, Geenen R, Kuijer R, van Middendorp H (2008) Psychological adjustment to chronic disease. Lancet 372 9634: 246–255. 18640461 10.1016/S0140-6736(08)61078-8

[40] Sherer R, Stieglitz K, Narra J, et al (2002) HIV multidisciplinary teams work: support services improve access to and retention in HIV primary care. AIDS Care 14 Suppl 1: S31–S44. 12204140 10.1080/09540120220149975

[41] Collins PY, Freeman M (2009) Bridging the gap between HIV and mental health services in South Africa. In: P. Rohleder, L. Swartz, S. Kalichman, and L. Simbayi (Eds.). HIV/AIDS in South Africa 25 Years On, Chapter 23: Psychosocial Perspectives. New York: Springer.

[42] Dodds S, Nuehring EM, Blaney NT, Blakley T, Lizzotte JM, et al (2004) Integrating mental health services into primary HIV care for women: the Whole Life project. Public Health Rep 119 1: 48–59. 15147649 10.1016/j.phr.2004.03.011 PMC1502259

